# Exploring the Limits of Cell Adhesion under Shear Stress within Physiological Conditions and beyond on a Chip

**DOI:** 10.3390/diagnostics6040038

**Published:** 2016-10-21

**Authors:** Melanie E. M. Stamp, Anna M. Jötten, Patrick W. Kudella, Dominik Breyer, Florian G. Strobl, Thomas M. Geislinger, Achim Wixforth, Christoph Westerhausen

**Affiliations:** 1 Chair for Experimental Physics 1, University of Augsburg, Augsburg 86159, Germany; melanie.stamp@physik.uni-augsburg.de (M.E.M.S.); anna.joetten@physik.uni-augsburg.de (A.M.J.); patrick.kudella@physik.uni-augsburg.de (P.W.K.); dominik.breyer@googlemail.com (D.B.); florian.strobl@physik.uni-augsburg.de (F.G.S.); thomas.geislinger@physik.uni-augsburg.de (T.M.G.); achim.wixforth@physik.uni-augsburg.de (A.W.); 2 Nanosystems Initiative Munich (NIM), Schellingstraße 4, Munich 80799, Germany; 3 Augsburg Center for Innovative Technologies (ACIT), Augsburg 86159, Germany

**Keywords:** cell adhesion, shear stress, pH, temperature, medical implants, microfluidics, lab-on-a-chip

## Abstract

Cell adhesion processes are of ubiquitous importance for biomedical applications such as optimization of implant materials. Here, not only physiological conditions such as temperature or pH, but also topographical structures play crucial roles, as inflammatory reactions after surgery can diminish osseointegration. In this study, we systematically investigate cell adhesion under static, dynamic and physiologically relevant conditions employing a lab-on-a-chip system. We screen adhesion of the bone osteosarcoma cell line SaOs-2 on a titanium implant material for pH and temperature values in the physiological range and beyond, to explore the limits of cell adhesion, e.g., for feverish and acidic conditions. A detailed study of different surface roughness *R_q_* gives insight into the correlation between the cells’ abilities to adhere and withstand shear flow and the topography of the substrates, finding a local optimum at *R_q_* = 22 nm. We use shear stress induced by acoustic streaming to determine a measure for the ability of cell adhesion under an external force for various conditions. We find an optimum of cell adhesion for *T* = 37 °C and pH = 7.4 with decreasing cell adhesion outside the physiological range, especially for high T and low pH. We find constant detachment rates in the physiological regime, but this behavior tends to collapse at the limits of 41 °C and pH 4.

## 1. Introduction

With an aging population, the need for medical implants is increasing rapidly. For example, a study from the Organization for Economic Co-operation and Development (OECD) in 2015 stated that per year 282 out of 100,000 citizen of the OECD countries will need knee or hip replacements [1]. Projections claimed that the number of arthroplasty surgeries will reach over 4 million annual procedures by the year 2030 [2], not even counting the numerous revision surgeries due to inflammatory complications.

After surgery, implants connect to the bone via the process of osseointegration. Therein, cells adhere to the implant to form a direct structural and functional connection between bone and implant. Important for osseointegration are cell-substrate contacts. This non-specific binding is a complex mechanism involving a variety of proteins and their interaction with materials. Most important are the extracellular matrix proteins (fibronectin, collagen, laminin, vitronectin), cytoskeletal proteins (actin, talin, vinculin), and integrins serving as membrane receptors [3]. The process of cell adhesion can be separated into three stages. First, the cell body attaches to the substrate (initial stage) by physico-chemical linkages, e.g., ionic forces and van der Waals forces, between the cell and the implant [4]. Second, the cell body flattens and spreads with adhesion sites formed by integrins to anchor the cell to a matrix or to adhesion molecules on other cells [5]. In the final phase the cells’ actin skeleton reorganizes and forms focal adhesion between the cell and its substrate [6]. Some focal adhesions disassemble while others enlarge and nucleate associated stress fibers, allowing non-muscular cells to withstand shear flow in blood streams [7]. Those stress fibers consist of bundles of actin filaments, mainly held together by the actin-crosslinking protein α-actinin. These stress fibers are of particular importance for cellular motility as fiber contractility helps endothelial cells to remain flat under flow and hence to reduce the experienced fluid shear forces [8].

After surgery, inflammation of the implant’s surrounding is caused for example by wear debris [9] or macrophage activated inflammatory cytokines that are released into the joint fluid, where they stimulate the differentiation of bone marrow cells into osteoclasts, leading to bone resorption [10]. The main reason for joint revision surgeries lies in the loosing of the implant material due to inflammation caused by such wear debris or bacterial infections [9,10,11]. Hereby infections and inflammatory reactions often result in changes of temperature, particularly feverish conditions [12], or decrease of the intercellular pH [13]. Depending on the extent of the inflammation, the implant will possibly fail, causing high costs for revision surgeries [11].

Thus, to ensure the reliability and durability of medical implants is of particular interest, and the strength of cell adhesion needs to be investigated in detail to understand the underlying processes. Several groups have already studied cell adhesion under static and dynamic conditions [14,15,16,17,18,19,20]. One of the first approaches to dynamically study cell adhesion used a rotating disk in a fluid above a substrate with adhered cells to generate a shear force field [14]. Using this setup, Weiss et al. demonstrated that shear stress strongly influences cell adhesion. However, due to its construction, the method does not allow for in situ observation of the cells, which hinders a detailed time-dependent investigation and high amounts of sample volume are needed in the setup’s recent development stage [15]. In contrast, the technique of surface acoustic wave (SAW)-induced streaming has been shown to enable the construction of much smaller setups to study cell adhesion with respect to shear flow [16,17,18]. Bussonnière et al. investigated the de-adhesion of cells directly from a piezoelectric substrate [16]. They demonstrated that SAW fluid actuation can be used to selectively detach and sort cells in a droplet. However, presuming the cells to adhere directly on the piezoelectric substrate prohibits the investigation of different substrate materials. Furthermore, the cells are not only exposed to shear flow but also to electric, thermal and mechanic influences of the propagating SAW which has been shown to alter the cells’ behavior [17]. We recently developed a SAW-activated system that works with small sample volumes and allows to decouple the single environmental influences from each other. It additionally provides the possibility to observe the cells in situ and to implement arbitrary substrate materials [18].

Here, we used this lab-on-a-chip system to study cell adhesion on titanium substrates as a model for medical implants and vary temperature, pH, and surface roughness to explore the limits of cell adhesion under various physiological conditions and beyond. Our experiments demonstrate an impaired cell adhesion above and below the regular physiological conditions of *T* = 37 °C and pH = 7.4. Inside the physiological range, cells withstand aggravated conditions, even when exposed to shear flow, which we use to actively add an external force working against cell adhesion. We only find significant changes in the cells’ adhesion for *T* > 41 °C, pH < 6.5 and pH > 8.0 in terms of a more than 50% increased cell detachment. Surface roughness also shows a clear effect on the cells ability to adhere and form bonds that effectively withstand shear forces. Although we find the highest adhesion on rough surfaces under static conditions, the optimum shifts to smoother surfaces if a shear flow is applied.

## 2. Materials and Methods

### 2.1. The De-Adhesion Number Investigator (DANI)

**Figure 1:**
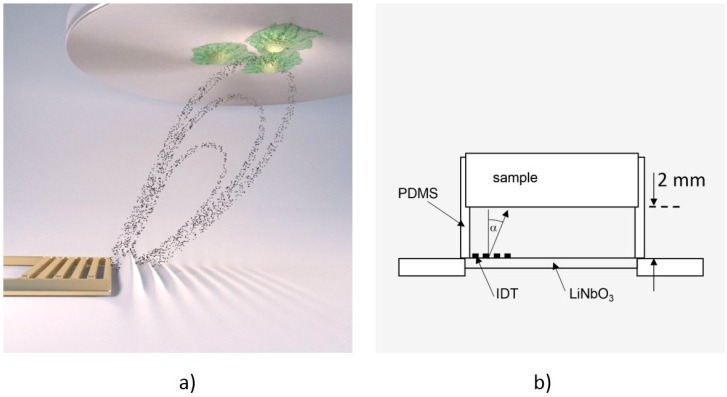
(**a**) Computer animation of the De-Adhesion Number Investigator (DANI) setup showing the acoustic streaming in the chamber towards the substrate with adhered cells (indicated by the black dots) that is generated by the interdigital transducer (IDT) (gold, comb-like structure) (by courtesy of C. Hohmann, Nanosystems Initiative Munich (NIM)); (**b**) Schematic drawing of the same setup. The IDT is located on the LiNbO_3_ chip inside the polydimethylsiloxane (PDMS)-chamber, which holds the substrate 2 mm above the chip. The fluid flow induced by the SAW is directed towards the cell substrate under an angle of α = 21°.

To measure cell de-adhesion under dynamic conditions, we employed the previously introduced microfluidic method De-Adhesion Number Investigator (DANI) [18] as illustrated in Figure 1. Described in brief, it consists of a cylindrical polydimethylsiloxane (PDMS) chamber with a volume of *V* ≈ 160 µL between a piezoelectric LiNbO_3_ chip and a circular substrate of arbitrary material (here: titanium) with adhered cells on top. We fastened the whole setup using a brass bridge, which is also thermally connected in order to heat the system using a heat bath. Here, the heat bath temperature is chosen about Δ*T* = 7 °C lower than the desired temperature. Together with the small SAW-generated temperature increase of the sample, this results in the temperatures given below. By applying a radio frequency signal to the interdigital transducer (IDT) on the piezoelectric substrate, surface acoustic waves were generated. These caused acoustic streaming leading to a fluid flow towards the cells under an angle of α = 21° relative to the surface normal [19].

### 2.2. Scanning Particle Image Velocimetry

We determined the average shear rate on the cells using scanning particle image velocimetry (SPIV) [20] to correlate the applied shear forces with cell detachment. In brief, SPIV is an automated acquisition and analysis approach based on the PIVlab toolkit by Thielicke [21,22,23]. It automatically scans an area larger than a single field of view and combines the multiple micro-particle image velocimetry (µPIV, for details see e.g., [24]) measurements to a single velocity field. It is able to repeat this process at several different heights and to correlate the data of the different height levels. This enables the semi-automated determination of three dimensional velocity fields in large sample areas. In this study, we added latex microbeads (diameter: 3 μm, Polybead^®^, Polysciences Inc., Hirschberg an der Bergstraße, Germany) as tracer particles to the fluid and recorded videos with a high-time resolution using a high-speed video camera (FASTCAM 1024PCI, Photron, Pfullingen, Germany). We determined the flow field in x–y direction in the plane as close as possible to the sample surface, since this is the relevant region to appraise its influence on the cells. For our experiments, we applied a power of *p* = 28 dBm to the IDT, which results in an average shear rate of $\dot{\gamma }=4314\;{\text{s}}^{-1}$. To investigate cell adhesion as a function of surface roughness and determine the ideal topography, we applied a milder shear flow by reducing the power to *p* = 25 dBm, which results in a shear rate of $\dot{\gamma }=2157\;{\text{s}}^{-1}$. This corresponds to a shear stress of about 2 Pa and is particularly of interest, as it is known from literature that, for example, endothelial cells show high response to very low shear stress between 0.1 and 0.8 Pa [25].

### 2.3. Sample Preparation

We obtained medical titanium alloy (Ti gr.5-ELI) from Valbruna Edel Inox GmbH, Nürtingen, Germany. The material was cut to discs (*r* = 5 mm, *h* = 2 mm) and sandblasted by Aesculap AG Tuttlingen, Germany. The initial surface roughness of these discs is *R*_q_ = 3.76 µm, which is determined by profilometric measurements (Dektak 8 Advanced Development Profiler, Vecco Instruments Inc., Oyster Bay, NY, USA). To investigate the influence of the surface roughness on cell adhesion, we polished the discs to yield seven different *R*_q_. Therefore, we embedded the substrates in Technovit^®^ 5071 (Heraeus Kunlzer GmbH, Wehrheim, Germany) and polished them using the auto-grinder and polisher AutoMet^®^ 250 (Buehler, Illinois Tool Works Inc., Esslingen am Neckar, Germany) with abrasive paper of different granulation (60, 320, 1000, 2500, and 4000), followed by a polycrystalline diamond polish (grain size 1 µm) and a chemo-mechanical polish (grain size 40 nm). We analyzed the surface topography with an atomic force microscope (NanoWizard^®^ AFM, JPK Instruments AG, Berlin, Germany ) and found *R*_q_ = 640 nm, 150 nm, 70 nm, 30 nm, 22 nm, 10 nm, and 2 nm, respectively. We cleaned all samples in an ultrasonic bath for 10 min in a 30% water in ethanol solution and finally sterilized them in an autoclave at 120 °C for 20 min.

### 2.4. Cell Culture Lines

We cultured SaOs-2 human bone osteosarcoma cells purchased from CLS (Cell Line Service GmbH, Eppelheim, Germany) using DMEM (Dulbecco’s modified eagle medium) with stable glutamine, 3.7 g/L NaHCO_3_ and 1.0 g/L d-glucose, adding 50 mL fetal bovine serum (FBS Superior), 10 mL HEPES 1 M, 5mL l-glutamine 200 mM, 5 mL MEM vitamins 100× (all reagents from Biochrom GmbH, Berlin, Germany), and 1 mL primocin (ant-pm-2, Invitrogen™, Thermo Fisher Scientific GmbH, Dreieich, Germany) in humidified air containing 5% CO_2_ at 37 °C. We harvested the confluent cells for our experiments, following the standard trypsinization procedure using 1 mL Trypsin/EDTA solution and PBS (w/o Ca^2+^, w/o Mg^2+^) (Biochrom GmbH, Berlin, Germany). By centrifugation and discarding of the supernatant with subsequent resuspension in media, we adjusted the cell density to 300,000 cells/mL.

### 2.5. Cell Adhesion and Fluorescence Imaging

We added 1 µL/mL of 1 µL calcein green acetoxymethyl fluorescent dye (Invitrogen™, Thermo Fisher Scientific GmbH, Dreieich, Germany) dissolved in 1 µL dimethyl sulfoxide to the cell suspension. In living cells, the acetoxymethyl (AM) esters are then removed by intracellular esterase and the whole dye molecule starts to fluoresce. This way, only living cells become visible in fluorescence microscopy. We incubated the cell suspension for 30 min, then transferred 200 µL of this suspension onto the substrate that is already inserted into the PDMS cylinder.

We incubated the cells for *t*_inc_ = 60 min allowing them to subside and adhere on the substrate. About 50% of the suspended cells adhere to the substrate under standard conditions (*T* = 37 °C, pH = 7.4, *R*_q_ = 3.76 µm). In the incubation step, we either set pH = 7.4 = const. and incubated at different temperatures *T* = 27, 33, 37, 39, 41, 42 and 47 °C, or we set the temperature to *T* = 37 °C = const. and incubated at different pH = 4.0, 4.5, 5.5, 6.5, 7.4, 8.0, 9.0, 10.0. To adjust the pH, we added HCl or NaOH to the culture medium to generate acidic or alkaline conditions, respectively. After incubation, we gently replaced the supernatant by a cell-free medium to remove the cells that did not adhere. Before starting the experiment, we then needed about 10 min to mount the device on the microscope and to connect the heating system.

**Figure 2:**
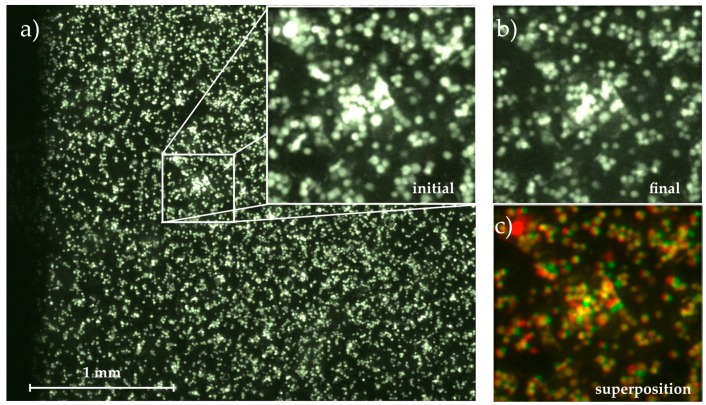
Micrograph of the adhered cells on the substrate with magnification of (**a**) initial state at *t*_0_ = 0 min; and (**b**) final state at *t* = 60 min; (**c**) Superposition of both images using the colocalization finder macro for ImageJ [26]. Cells remaining fixed throughout the whole measurement are colored in yellow, detached cells are colored in red and moved or newly adhered cells are green.

Using an inverted fluorescence microscope (Axiovert 200M, Zeiss) equipped with a 2.5× objective and a digital camera (Orca 5G, Hamamatsu Photonics Deutschland GmbH, Herrsching am Ammersee, Germany), we observed an area of *A_tot_* = 3.48 mm × 2.65 mm = 9.22 mm^2^ (compare Figure 2). We recorded micrographs of the cells before applying a shear flow (static measurement, $t=0\text{ min}=t_{0}$), and one micrograph every 5 min with applied shear flow for a period of 60 min ($0\leq t_{i}\leq 60\text{ min}$) and quantified the amount of adhered cells as the fraction of the total field of view covered by cells

(1) $$A_{c,t}=A_{covered}\cdot A_{tot}$$

The static measurement thus corresponds to the initial value of $A_{c,t_{0}}=A_{c,0\;min}$.

To determine *A_c,t_* time-dependently, we converted the recorded micrographs into an 8-bit black and white format using the software ImageJ [26]. For each experiment, we set an individual but constant threshold to distinguish adherent cells from the background. The intensity is then inverted and the area covered with cells (black) on the free area (white) is quantified using the ImageJ particle analysis function and correlated with the starting value $A_{c,t_{0}}$ to determine the detached cells under flow.

Comparing the following images to this initial state, we determined the migration of cells on the substrate (area covered with cells at $t_{i}>0\text{ min}$ but not at *t*_0_) and their detachment (area covered with cells at *t_0_* but not at $t_{i}>t_{0}$) time-dependently, ending up with $A_{c,\left(t\;=\;60\text{ min}\right)}=A_{c,60\;min}$. Figure 2 visualizes this image analysis process. The magnified images show the initial and final state as well as their superposition created with the colocalization finder macro for ImageJ [26]. In the superposition, the yellow cells remain at their positions throughout the whole experiment, while spots where cells have been detached are labelled with red and cells that appear in the final image but not in the initial one and thus must have moved on the substrate are shown in green.

## 3. Results and Discussion

Using DANI, we examined the de-adhesion of SaOs-2 human bone osteosarcoma cells from titanium substrates that are commonly used as implant material and investigated the cell detachment under static and dynamic conditions. First, static measurements delivered information on gravity induced cell detachment as well as artifacts like bleaching of the fluorescent dye. Second, we varied either the temperature, the pH or the surface roughness of the substrate while keeping the respective other two parameters constant. Finally, we applied shear stress on the cells to determine its influence on the de-adhesion under various conditions. The SAW-induced shear rate was primarily used not to simulate biologic flow conditions inside the body, as we explain below, but to create a measure for examining and characterizing the ability and strength of cell adhesion with a well-defined hydrodynamic force.

### 3.1. Time-Dependent Cell Detachment

We used the standard substrate with *R*_q_ = 3.76 µm to investigate the time-dependency of cell adhesion under static and dynamic conditions for *T* = 37 °C and pH = 7.4.

In a first step, we determined the gravity-induced cell detachment and characterized the systematic influence of bleaching of the fluorescence stain. Detachment of cells by gravity appears in our system since the cells adhere to the lower side of the substrate serving as a lid of our flow chamber (compare Figure 1). Thus, a fraction of the cells detaches over time by falling from the substrate. Bleaching occurs for calcein green AM since it is constantly metabolized by living cells. Due to the decreasing fluorescence signal over time the thinner fringe areas of the cells happen to fall below the intensity threshold.

**Figure 3:**
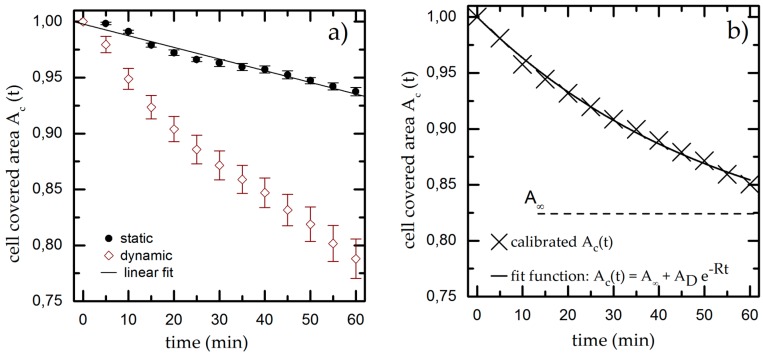
(**a**) Cell covered area over time under static and dynamic conditions for *T* = 37 °C, pH = 7.4 and *R*_q_ = 3.76 µm. We fit a linear function to the static results (black line) and use it as calibration baseline to decouple the shear-induced detachment from the effects of gravity and bleaching. The data points and error bars show the mean and standard deviation respectively from *n* ≥ 5 measurements; (**b**) Dynamic measurement from (**a**) normalized to the linear fit extracted from the static experiments.

We quantify both effects by determining the cell covered area *A_c,t_* in time steps of 5 min for up to 60 min under static conditions. We normalize *A_c,ti_* to the initial value for the cell covered area *A_c,t_* = 31% ± 3% and plot it over time, as shown for a typical example in Figure 3a. We find a linear decrease of *A_c,t_* down to *A*_*c*,60 min_ = 0.94 ± 0.03 * *A*_*c*,0 min_ meaning that gravity and bleaching result in a decrease of *A_c,t_* of approximately 6% within 60 min. The experiments are repeated at least five times to compensate for deviations of the initially seeded cell number and cell viability.

The results of the dynamic experiments reveal the influence of shear on cell adhesion and are plotted in Figure 3a. We also find an exponential decay for *A_c,t_* down to *A*_*c*,60 min_ = (0.79 ± 0.03) * *A*_*c*,0 min_. To decouple gravity and bleaching from the influence of shear, we now normalize the results of the dynamic experiment using the linear fit function from the static measurement as baseline (used in all later dynamic measurements). The resulting graph (as shown exemplarily in Figure 3b) finally shows only the cell detachment due to shear forces.

We fit an exponential function to the normalized dynamic measurement, which is given by

(2) $$A_{c,t}=A_{\infty }+\left(1-A_{\infty }\right)*e^{-Rt}$$

where *A* represents the cell covered area for *t→∞*, $A_{D}:\;=\;\left(1-A_{\infty }\right)$ the fraction of detached cells and *R* as detachment rate to determine the cell covered area *A_c,t_* at given time *t*. The calibrated, shear-induced decay now leads to *A*_*c*,60 min_ = (0.85 ± 0.03) * *A*_*c*,0 min_.

### 3.2. Influence of Temperature, pH and Surface Roughness on Cell Adhesion

**Figure 4:**
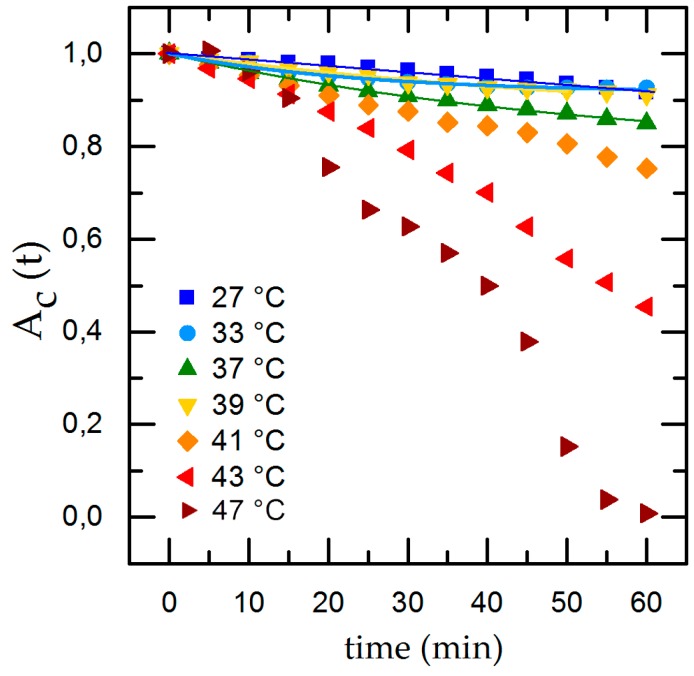
Temperature-dependent cell detachment with time and exponential fit for detachment rate in the physiological range of 27 °C ≤ *T* ≤ 39 °C.

**Figure 5:**
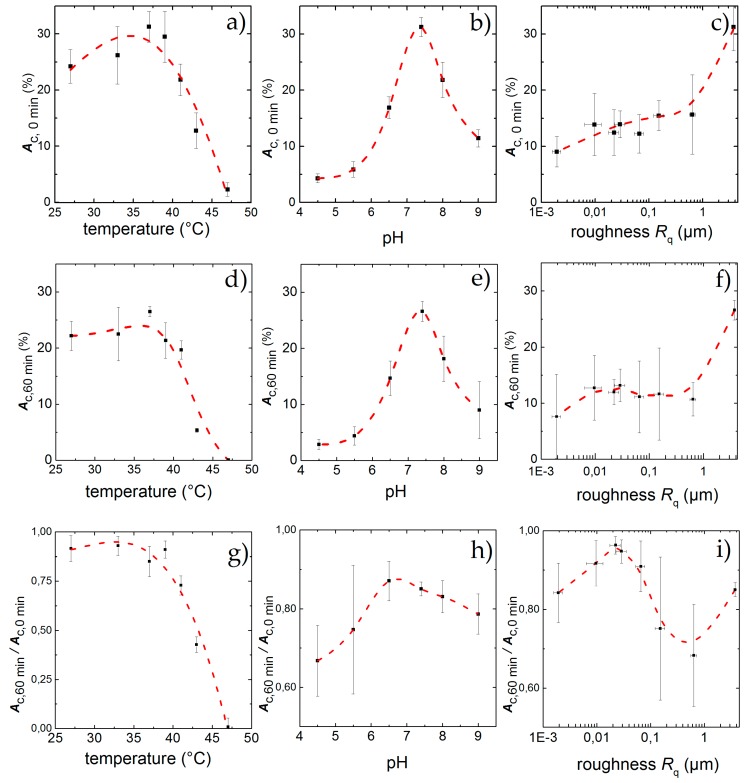
Percentage of area covered with cells after an incubation time of 60 min under standard culture conditions but for different temperatures (**a**), pH (**b**) and surface roughness (**c**); Physiological standard conditions of *T* = 37 °C and pH = 7.4 show the highest adhesion *A_c_*,_0 min_ while increasing roughness leads to higher *A*_*c*,0 min_. After applying shear flow for 60 min cell detach leading to a remaining adhesion *A*_*c*,60 min_ for different temperatures (**d**), pH (**e**) and surface roughness (**f**). While the effect of low temperatures hardly effect the cells, high temperatures and deviations from pH = 7.4 lead to strong detachment. For the influence of the topography, a peak in cell adhesion appears for *R*_q_ = 22 nm, although the highest final adhesion can be found for *R*_q_ = 3.76 µm. *A*_*c*,60 min_/*A*_*c*,0 min_ denotes the fraction of the field of view that is covered with cells after 60 min exposure to shear flow. Similar to static conditions, the influence of temperature (**g**), and pH (**h**) inside the physiological range changes *A*_*c*,60 min_/*A*_*c*,0 min_ only slightly, whereas high temperatures and low pH exhibit a strong effect; *A*_*c*,60 min_/*A*_*c*,0 min_ as a function of surface roughness (**i**) shows a maximum at *R*_q_ = 22 nm. The data points and error bars show the mean and standard deviation respectively from *n* ≥ 5 measurements and the dashed lines are guides to the eye.

As mentioned above, inflammatory conditions can lead to changes in temperature and pH. Therefore, we examined in the following cell detachment under static and dynamic conditions and vary temperature, pH as well as the surface roughness. We studied their respective influence and find the results shown in Figure 4 and Figure 5. We individually compared the static result in terms of the initial adhesion *A*_*c*,0 min_ as well as the dynamic results in terms of the final adhesion *A*_*c*, 60 min_ and the normalized final adhesion *A*_*c*,60 min_/*A_c_*_,0 min_ as a function of temperature (Figure 5a,d,g), pH (Figure 5b,e,h) and surface roughness (Figure 5c,f,i), respectively.

• Temperature

The normal temperature of the human body is 37 °C and can vary from 33 °C during therapeutic hypothermia up to 42 °C during high fever, which is a reaction of the body to inflammation or infection [12]. Therefore, we chose various temperatures within and beyond this physiological range to explore the cell adhesion and its limits. During incubation and the experiments, we set the temperature to *T* = 27, 33, 37, 39, 41, 43, and 47 °C, respectively, while keeping the pH and surface roughness constant at pH = 7.4 and *R*_q_ = 3.76 µm.

The results under static conditions are shown in Figure 5a. We find an asymmetric behavior of *A*_c_ over *T* with a clear maximum of *A*_*c*,0 min_ = 31% ± 3% for *T* = 37 °C. For lower temperatures, *A*_*c*,0 min_ decreases only slightly to *A_c,_*_0 min_ = 26% ± 5% at *T* = 33 °C at the lower end of the physiological range. Even for a temperature as low as *T* = 27 °C, *A*_*c*,0 min_ = 24% ± 3% and thus about 78% of its maximum value at 37 °C, while it decreases strongly for *T* ≥ 37 °C, down to *A*_*c*,0 min_ = 22% ± 4% for *T* = 41 °C, just below the upper physiological limit. A significant change can then be observed for *T* = 43 °C, where the cell-covered area falls to *A*_*c*,0 min_ = 13% ± 5% (corresponds to 32% of the maximum). The highest temperature *T* = 47 °C only shows traces of cell adhesion with *A*_*c*,0 min_ = 3% ± 1%.

The results with applied shear flow for *t* = 60 min are shown in Figure 5d. Here we find a maximum *A*_*c*,60 min_ = 27% ± 3%. Low temperatures show a final adhesion of *A*_*c*,60 min_ = 23% ± 3% for *T* = 27 °C and *A*_*c*,60 min_ = 26% ± 5%. Even for 39 °C and 41 °C with *A*_*c*,60 min_ = 21% ± 3% and *A*_*c*,60 min_ = 19% ± 2% there is no significantly increased detachment under shear flow. Only at high temperatures with *T* ≥ 43 °C the final adhesion drops down to *A*_*c*,60 min_ = 5% ± 1%. For *T* = 47 °C no adhered cells can be found after shear flow exposure.

To compare the individual changes during shear flow exposure we normalize the time-dependent cell adhesion to the initial adhesion and show the results with applied shear flow in Figure 4. Under dynamic conditions we find an exponential decrease of the cell covered area *A_c,t_* with time. Applying our rate model (Equation (2)) for temperatures inside the physiological range (33 °C ≤ *T* ≤ 41 °C), we can calculate the detachment rate *R* and $A_{\infty }$, whereas going to extreme conditions (*T* = 27 °C and *T* ≥ 43 °C), the rate model collapses. At *T* = 27 °C we find only minor cell detachment in the beginning and an increase of cell detachment at *t* = 40 min as well as a final cell covered area of $A_{\infty }=$ 0. This is no reasonable result and is due to the flat slope and the concomitant large error of the fit. At *T* = 43 °C *A_c,t_* shows an almost linear decrease and, after *t* = 40 min, an abrupt decline. For *T* = 47 °C we find the same effect but with a stronger decline and no remaining cells on the substrate for *t* = 60 min. The abrupt change of the detachment rate prohibits to fit Equation (2) to the data.

Thus, to be able to quantitatively discuss and classify our results of the dynamic experiments, we use the final adhesion *A*_*c*,60 min_ and normalized final adhesion *A*_*c*,60 min_/*A*_*c*,0 min_ instead of $A_{\infty }$. Doing so, we find the maximal cell adhesion for *T* = 33 °C (*A*_*c*,60 min_/*A*_*c*,0 min_ = 0.92 ± 0.09) and an equally high value of *A*_*c*,60 min_/*A*_*c*,0 min_ = 0.91 ± 0.6 for *T* = 27 °C. Both values are higher than the one for *T* = 37 °C (*A*_*c*,60 min_/*A*_*c*,0 min_ = 0.85 ± 0.07), although the values overlap clearly and are about equal to *A*_*c*,60 min_/*A*_*c*,0 min_ = 0.92 ± 0.05 for *T* = 39 °C. Obviously, temperatures just below the physiological range and close to the normal body temperature do not have significant impact on cell adhesion. However, for temperatures of *T* ≥ 41 °C, which approach the physiological limit, we find a decrease of *A*_*c*,60 min_/*A*_*c*,0 min_ down to *A*_*c*,60 min_/*A*_*c*,0 min_ = 0.72 ± 0.04. Here, the detachment rate is increased by a factor of 1.2 compared to *T* = 37 °C. Exceeding the physiological limits (*T* = 43 °C) leads to even higher detachment rates, leaving only a fraction of 40% of the initially attached cells with *A*_*c*,60 min_/*A*_*c*,0 min_ = 0.45 ± 0.05 on the substrates (about half of the according value for *T* = 37 °C). For *T* = 47 °C, no cells are left on the substrate after 60 min. This behavior of *A*_*c*,60 min_/*A*_*c*,0 min_ as function of temperature is qualitatively similar compared to the static measurements (see Figure 5a,g).

To our best knowledge no detailed studies on temperature-dependent cell adhesion are available to date, thus, we compare our results to studies on the influence of temperature on cell viability and protein stability, which both contribute to cell adhesion. The rather small impact of temperatures below the physiological standard can be understood by the fact that cell vitality is slowed down significantly, but is not entirely interrupted. Additionally, proteins remain operational at such low temperatures. This is supported by a long-term study, which even revealed that culturing cells at lower temperatures hardly effects their proliferation [13]. Although the metabolic rates of the glucose uptake and lactate production are lowered, the growth rate at *T* = 33.5 °C was shown to be only 8% lower than at 37 °C [13]. Even lower cultivation temperatures of *T* < 30 °C have been proposed as a means to control cell proliferation [27], stating that a controlled growth rate can be used to inhibit the lactate dehydrogenase (LDH) activity which has been shown to directly affect the cell’s viability [28]. Combining these studies with our findings, we find that the minor initial cell coverage at *T* = 33 °C can even promote cell adhesion under flow. These studies support our assumption that small deviations in temperature from 37 °C do not affect cell adhesion.

Our experiments reveal that medium temperatures of 27 °C ≤ *T* ≤ 41 °C provide excellent environmental conditions for cells to adhere and withstand shear flow. The time-dependent detachment shown in Figure 4 indicates a change of the detachment behavior at higher temperatures (*T* ≥ 43 °C) and even total detachment after 60 min for *T* = 47 °C. We propose this behavior to be caused by cytotoxic effects due to the high temperatures. This conclusion is supported by medical studies on hypothermal treatment in cancer therapy, focusing on cell exposure to thermal dose [29]. Here, a dose dependency of thermal cell killing with temperature and exposure time was found. The study found nearly no changes in cell viability at *T* = 41 °C even after 6 h, but heat-induced cytotoxicity for *T* > 42.5 °C [30].

The faster cell detachment for higher temperatures (*T* ≥ 41 °C) can be understood by relating it to the temperature-induced denaturation of proteins at high temperatures, leading to a break of the bonds between cell and substrate or even cell death [31]. Furthermore, thermal changes effect the fluidity and stability of cellular membranes and impede the function of transmembrane transport proteins and cell surface receptors, leading to cytotoxic influences and eventually cell death [32]. Thus, the cytotoxic effect of hyperthermia is mainly based on denaturation of cytoplasmic and membrane proteins [30]. Here, most important for cell adhesion is the thermal destruction of integrins [33] and the cytoskeleton [34]. We finally propose the cytotoxic effect of temperature to be the reason for the abrupt increase of cell detachment over time at higher temperatures and for their complete detachment at *T* = 47 °C.

Considering medical implants, our results suggest successful osseointegration under flow conditions for temperatures below 41 °C. For higher temperatures that may occur due to inflammations, cell adhesion and in turn osseointegration becomes problematic.

• pH

The physiological pH of intercellular body fluids is around pH = 7.4. Although changes between 6.8 ≤ pH ≤ 7.8 may occur during infections, inflammation or medication, cells are still able to survive under such conditions [35]. However, in some severe infections the intercellular pH can even drop down to pH = 6.0 [36].

In this study, we varied the pH of the nutrient medium in the range of 4.0 ≤ pH ≤ 10.0 as described in the methods section while keeping the temperature and surface roughness constant at *T* = 37 °C and *R*_q_ = 3.76 µm.

Again, we find our rate model (Equation (2)) to be applicable only within the physiological range and use again *A*_*c*,60 min_ to quantify the cell adhesion under dynamic conditions.

Figure 5b displays the results for the static case in terms of *A*_*c*,0 min_ as a function of the pH. Qualitatively, we find an asymmetric distribution of *A*_*c*,0 min_ with a stronger cell detachment for acidic pH than in the alkaline range. A more quantitative analysis shows a maximum of *A*_*c*,0 min,max_ = 31% ± 3% at pH = 7.4. In the acidic direction, we observe a strong decrease at the border of the physiological range (pH = 6.5) down to *A*_*c*,0 min_ = 17% ± 3% = 0.54 * *A*_*c*,0 min,max_ . For even lower pH, the final cell adhesion decreases strongly and we find only *A*_*c*,0 min_ = 4% ± 1% for pH = 4.5. As a limit of the cells’ adhesion capability, we identify pH = 4, where the cells do not adhere to the substrate at all. In an alkaline environment (pH = 8.0) we find *A*_*c*,0 min_ = 22% ± 4% = 0.7 * *A*_*c*,0 min,max_ . A stronger alkaline pH of 9.0 results in *A*_*c*,0 min_ = 11%. ± 2% = 0.35 * *A*_*c*,0 min,max_ and we find the limit for cell adhesion in alkaline media to be for pH ≥ 10.

After applying shear force for a time period of 60 min we find a residual adhesion of *A*_*c*,60 min_ = 27% ± 3% for pH = 7.4 as depicted in Figure 5e. For the acidic environment we find *A*_*c*,60 min_ = 15% ± 3% for pH = 6.5 and only about *A*_*c*,60 min_ = 3% ± 1% remaining cells at the lower end with pH = 4.5. Deviations from ideal pH towards alkaline milieu result in stronger detachment of cells, leaving a remaining *A*_*c*,60 min_ = 17% ± 3% for pH = 8 and *A*_*c*,60 min_ = 8% ± 5% for pH = 9.

The dynamic measurements normalized to the initial adhesion (depicted in Figure 5h) indicate a shift of the maximum value of initial covered area from pH = 7.4 (*A*_*c*,60 min_/*A*_*c*,0 min_ = 0.85 ± 0.03) to pH = 6.5 with *A*_*c*,60 min_/*A*_*c*,0 min_ = 0.87 ± 0.07, although the standard deviation of these values overlap strongly. To the alkaline side, we observe only a slight decrease of cell adhesion down to *A*_*c*,60 min_/*A*_*c*,0 min_ = 0.79 ± 0.07 at pH = 9. In contrast, towards the acidic side, we find the cell detachment to be increased (*A*_*c*,60 min_/*A*_*c*,0 min_ = 0.67 ± 0.09 for pH = 4.5). However, the effect of these extreme deviations of the pH from its optimum is less pronounced than the influence of temperature.

Both our static and dynamic measurements indicate that an acidification of the environment leads to a stronger inhibition of cell adhesion than an alkalization and that cells show much stronger resistance to pH changes even outside the physiological range compared to temperature deviations. This behavior can be explained by the cells’ strategies to regulate their intracellular pH using ion-exchange mechanisms [37]. The main challenge faced by pH regulatory mechanisms is to relieve the cell of excess protons, originating from accumulation of metabolically generated acids and from passive diffusion of H^+^ into the cell due to the internally negative membrane potential [38]. Therefore, cells have evolved several methods for pH regulation, e.g., Na^+^/H^+^ antiporters and Na^+^ dependent HCO_3_^−^/Cl^−^ exchanger for acidic regulation, as well as the Na^+^ independent HCO_3_^−^/Cl^−^ exchanger in alkaline pH [39]. It is expected that the cell metabolism is affected by intracellular (cytoplasmic) pH, which is influenced by Na^+^/H^+^ exchangers acting on the enzyme phosphofructokinase, the rate-controlling enzyme in the glycolytic pathway. Irreversible cytoplasmic acidification starts at pH 6.8 and below as here the pH leads to an enzyme inactivity [38] and a decrease in adhesion molecule expression which is responsible for unspecific binding processes [40].

Regarding our results, we consider these pH regulations to be active in the physiological range, as we can find the strongest cell adhesion for pH = 7.4. Under static conditions, we find *A*_*c*,0 min_ decreasing as the expression of adhesion molecules will decline. Nevertheless, the unspecific binding mechanisms are still able to withstand shear flow.

A study conducted by Serrano et al. [40] investigated the pH-dependency of adhesion molecule expression on endothelial cells under static conditions with 6.5 ≤ pH ≤ 8.4. They find vascular cell adhesion molecule expression to not exhibit a pH-dependency, whereas a strong decrease of intercellular adhesion molecules, responsible for specific binding, exists with pH deviations from 7.4. As we focus on cell-substrate interactions, these results confirm our findings for non-specific binding. Therefore, we can state that cell adhesion for small changes in pH is hardly affected, as in this range the pH regulating mechanism are active.

Concerning cell adhesion of human cell lines under dynamic conditions, there exist no other studies focusing on the influence of pH variations. However, our results are in good qualitative agreement with Crouch et al. [41], who studied the effect of pH on the adhesion of baby hamster kidney (BHK) cells to glass under static conditions, finding the strongest adhesive force at pH = 7.6 and a similar trend with pH as in our experiments.

A long term observation of the cells in our actual approach is not possible, due to the bleaching of the Calcein Green. In future studies, using green fluorescent protein (GFP)-transfected cell lines may open new possibilities and allow for studies of long term reaction of cells to extreme pH under flow.

• Surface Roughness

Apart from temperature and pH, surface roughness is an important factor for controlling cell adhesion, especially for a desired rapid and successful osseointegration of implants and their long-term stability. Bone bioactivity involves physicochemical surface reactions and cellular mechanisms. The surface composition and structure of bones influence the kinetics of protein adsorption, leading to bond formation and spreading [42]. Thus, the surface morphology obviously impacts cell adhesion. We here analyze the role of surface roughness on cell adhesion by preparing a set of samples of different surface roughness as described in the methods section. In these experiments, the temperature and pH were kept constant at *T* = 37 °C and pH = 7.4 for all samples.

Figure 5c shows the influence of the surface roughness on the initial cell adhesion under static conditions. A maximum in cell density (*A*_*c*,0 min_ = 31% ± 3%), is reached at the sandblasted surface roughness of *R*_q_ = 3.76 µm, whereas the use of polished surfaces leads to lower cell adhesion of *A_c_*_,0min_ ≈ 15%, nearly independent of the surface roughness.

In contrast, under dynamic conditions, *A*_*c*,60 min_ shows a maximum of adherent cells at *R*_q_ = 22 nm (*A*_*c*,0 min,max_ = 14% ± 4%) as can be seen in Figure 5f and therefore a remaining fraction of *A*_*c*,60 min_/*A*_*c*,0 min_ = 0.96 ± 0.04 normalized to the initial adhesion (see Figure 5i). Above and below this value, we find a decrease of *A*_*c*,60 min_. However, we still find *A*_*c*,60 min_ = 8% ± 7% and respectively *A*_*c*,60 min_/*A*_*c*,0 min_ = 0.84 ± 0.08 for the smoothest surface with *R*_q_ = 2 nm. Eying the rough surfaces, we find a decline in remaining cells with increasing roughness with a minimum for *R*_q_ = 640 nm with *A*_*c*,60 min_ = 10% ± 3% and respectively *A*_*c*,60 min_/*A*_*c*,0 min_ = 0.68 ± 0.12 = (0.8 ± 0.14) * *A*_*c*,0 min,max_. For the untreated standard sample with *R*_q_ = 3.76 µm the maximum in adhered cells can be found again with *A*_*c*,60 min_ = 27% ± 3%. Although normalized to the initial adhesion, the detachment increases to *A*_*c*,60 min_/*A*_*c*,0 min_ = 0.85 ± 0.03 =(0.90 ± 0.03) * *A*_*c*,0 min,max_ and therefore shows a medium adhesion behavior compared to all substrates.

The reason for this behavior can be found in the interactions of cells with the substrates that are mediated by subcellular microstructures resulting in focal attachment [43]. The focal contacts are anchored in the cell’s cytoskeleton and permit the cell to bind to an extracellular matrix via integrin [44]. For high rates of integrin binding, these focal contacts are of a certain length to efficiently promote adhesion and a maximum contact area of the cell with the substrate [6]. Additionally, cells align with the substrate’s topography in a way to minimize distortions of their cytoskeleton [45]. Therefore, variations in the substrate’s microtopography directly affect the cellular adhesion to an implant. However, the preferred roughness varies between different cell lines. Osteoblast-like cells attached better to rougher surfaces, whereas fibroblasts prefered smoother surfaces [44]. We here find that SaOs-2 cells show an osteoblast-like behavior with a local maximum at *R*_q_ = 22 nm.

We compare our results to Huang et al. [46], who studied the adhesion of osteoblast-like cells under static conditions on a similar set of surfaces. They find that the cell attachment is highly influenced by surface roughness in the range of 50 nm ≤ *R_a_* ≤ 1.20 µm, with *R_a_* = 150 nm showing the best cell adhesion and spreading compared to smoother (*R*_a_ ≤ 70 nm) or rougher (*R*_a_ ≥ 330 nm) substrates. We, too, see a strong increase in cell adhesion with increasing roughness up to *R*_q_ = 150 nm, but no further increase for 640 nm. Therefore, we conclude that an appropriate surface roughness can produce beneficial mechanical interlocking at the initial adhesion stage and foster further cell adhesion with its ideal spacing at *R_q_* = 150 nm [47].

In contrast, under dynamic conditions we find increased detachment with increasing surface roughness *R*_q_. This observation can be explained by shear flow-induced activation of focal adhesions, leading to an enhancement of stress fibers [7]. Integrin receptors function as mechano-sensors and induce an activation on signaling proteins which act on the cytoskeletal anchorage [48]. It has been reported that surface topography correlates with binding of focal adhesion and thus formation of stress fibers. Ideal spacing for focal contacts was found to be 30 nm [6], which is in good agreement with our results that show maximal cell adhesion under dynamic conditions for *R*_q_ = 22 ± 5 nm. Along the same lines, we compare our results to the ones reported by Cavalcanti-Adam et al. [49], who used nano-patterned surfaces to investigate the influence of ligand spacing to cell adhesion under static conditions. They found the initial attachment of cells to be independent of the surface, but for the formation of stable focal adhesion and persistent spreading they found a critical ligand density (interligand spacing < 70 nm). Our findings indicate that we see a similar effect, although we are varying the sample topography and not the ligand density.

Combining those results, we conclude that surface roughness in the range of filopodia size (≈70 nm) should offer the highest adhesion strength under dynamic conditions for relatively smooth surfaces. For increased surface roughness in the range of several microns, additional effects like shielding may appear. These results indicate that if for other reasons smooth implant surfaces are necessary, the roughness should be designed to be in the range of 22 ± 5 nm to offer good premises for osseointegration.

The existence of an optimum is intuitively plausible: a smooth, polished surface does not provide any contact points for a cell to form strong bonds, while a very uneven profile requires strong bending of the cell’s membrane to attain a large contact area. A moderate roughness could comply with injured tissue as small fractures in bone material, where growth and adhesion of bone cells is essential.

Based on these findings we claim that the introduced method by characterization of cell adhesion using shear flow could be used as an indicator for the examination of novel materials regarding their ability for osseointegration. Of course, this needs in-depth studies to correlate cell adhesion in vitro under flow with in vivo studies of the same materials. In this sense, our data on titanium implants would predict sufficient osteoblastic cell adhesion even when exposed to deviations from standard body conditions but inside the physiological possible range. A direct conclusion from the results presented here on cells in intraosseous blood flow cannot be drawn. To our best knowledge there is a lack of reports in literature on appearing intraosseous blood flow conditions regarding vessel geometry and flow rates. The best information on this is reported for overall intraosseous flow rates in animal tissue per mass [50,51,52]. Thus, a precise estimation of acting hydrodynamic shear forces on osteoblasts in vivo cannot be given to date.

## 4. Conclusions and Outlook

Summing up, our static experiments show the strongest adhesion of SaOs-2 cells on titanium substrates with *R*_q_ = 3.76 µm under physiological standard conditions of *T* = 37 °C and pH = 7.4. Applying shear flow reveals an even more pronounced decrease of cell adhesion for deviations from these conditions. High temperatures and low pH are especially critical. Under extreme conditions we find our rate model collapses due to cell death during the experiment. Regarding surface roughness, we found a local optimum of adhesion at an average roughness of *R_q_* = 22 nm. Future studies should also account for the influence of cell density on the de-adhesion rate, since, for example, cells are shielded from the flow by cells positioned further upstream or adhesive forces between cells may appear. A quantitative correlation between local shear rates and time-dependent detachment of cells might give further insight into the shear sensitivity of arbitrary cell-substrate-combinations. Interesting systems thereby include novel metal ion releasing implant materials [53] and osteoblasts. On the other hand, cell biologists may apply the DANI-setup to study the internal response of adaptive processes of adherent cells to shear flow. Such processes are, for example, the mobility of adhesive bridges, the creation of reactive oxygen species, the structure and the orientation of the cytoskeleton as well as gene expression. A comparison of the results with in vivo experiments could then highlight the biological relevance of such studies.

## Abbreviations

The following abbreviations are used in this manuscript:

DANI	De-Adhesion Number Investigator
SPIV	scanning Particle Image Velocimetry
SAW	Surface Acoustic Waves
IDT	Inter Digital Transducer
